# Pathways and challenges towards a complete characterization of microgels

**DOI:** 10.1038/s41467-020-17774-5

**Published:** 2020-09-04

**Authors:** Frank Scheffold

**Affiliations:** Department of Physics, University of Fribourg, Chemin du Musée 3, 1700 Fribourg, Switzerland

**Keywords:** Polymers, Colloids, Gels and hydrogels, Super-resolution microscopy

## Abstract

Due to their controlled size, sensitivity to external stimuli, and ease-of-use, microgel colloids are unique building blocks for soft materials made by crosslinking polymers on the micrometer scale. Despite the plethora of work published, many questions about their internal structure, interactions, and phase behavior are still open. The reasons for this lack of understanding are the challenges arising from the small size of the microgel particles, complex pairwise interactions, and their solvent permeability. Here we describe pathways toward a complete understanding of microgel colloids based on recent experimental advances in nanoscale characterization, such as super-resolution microscopy, scattering methods, and modeling.

## Abstract (web summary)

Despite their widespread use, many fundamental questions about the internal structure of microgels are still open. Here the authors describe several pathways toward a complete understanding of microgel colloids based on recent experimental advances in nanoscale characterization.

## Introduction

**Fig. 1:**
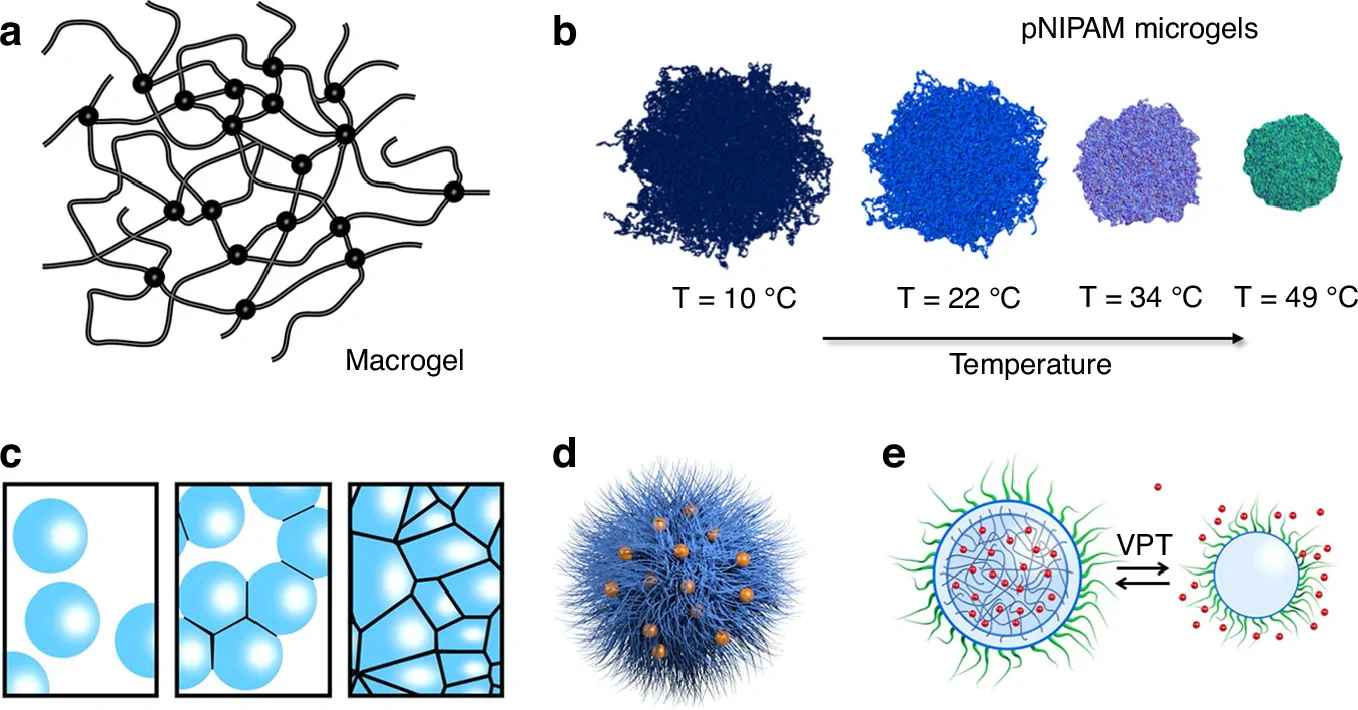
Polymer microgel morphology, swelling, packing, and functionality. **a** Sketch of a macroscopic polymer gel with a statistically homogeneous distribution of permanent or reversible cross-links. In a good solvent, the gel is swollen, and the degree of swelling is controlled by a balance between the mixing entropy, favoring swelling, and the elastic penalty when stretching the polymer chains between cross-links^21,36^. **b** Volume-phase transition (VPT) of stimuli-responsive microgel particles. Microgels are colloidal-sized polymer gels, typically with a radius of 1 μm or less, where the crosslinker density is usually decaying from the center to the periphery^1,27^. The polymers in the network can be neutral (nonionic) or carrying charged groups. The images shown are adapted with permission from ref. ^75^ (Copyright American Chemical Society, 2017) and depict the results from atomistic computer simulations of neutral microgels with a cross-link gradient and modeled at different temperatures. The polymer represents pNIPAM, poly-N-isopropylacrylamide, which has a lower critical solution temperature (LCST) in water of *T*_LCST_ ≃ 32 °C^81^. In agreement with experiments, the simulations show that below *T*_LCST_, the microgel colloid is highly swollen while above it collapses. **c** Swollen microgels are soft and compressible spheres, in osmotic equilibrium with the solvent phase and other microgels. At higher concentrations, particles pack densely and form a crystal or amorphous phase. With increasing total polymer concentration, the microgel particles become compressed, facet, and shrink^4^, details of which have a profound impact on the effective pairwise interactions^91^ and the macroscopic flow properties^25^. **d** By attaching metallic nanoparticles, such as gold, or by encapsulating pharmaceutically active substances, functional microgels can be obtained^12,13^. The mean distance between plasmonic nanoparticles can be probed optically, and microgels with the appropriate polymer chemistry can act as sensors^10,11^. **e** Triggering the VPT by temperature, pH, or optically can be used to expel molecules in a biological environment in a controlled way and at a targeted site. The graphic, adapted with permission from ref. ^24^ (Copyright 2013 Wiley Periodicals, Inc.), shows a sketch of a microgel particle filled with small active molecules (red dots) and decorated with polyethylene glycol (green hairs) to improve biocompatibility.

The term “microgel” usually refers to a cross-linked polymer network of colloidal size that is swollen in a good solvent and has a diameter between  ~100 nm and several micrometers^1–3^ (Fig. 1). Colloidal hydrogels are a common subtype where the continuous phase is water. Often, but not always, these networks exhibit conformational changes in response to changes in their environment, such as the solvent composition or temperature^1,4,5^. The sensitivity to external stimuli, such as temperature, pH, or solvent conditions, is an essential feature of many microgels, in particular for the most widely studied type of microgels, based on pNIPAM (poly-N-isopropylacrylamide) and its derivatives^1,6^ (Fig. 1b). Here, we consider homogeneous suspensions of these particles at different densities from the dilute to the highly packed regime, as well as microgels adsorbed at surfaces. It is also possible to synthesize larger, millimeter-sized, gel particles^7^, or to study two-dimensional assemblies at an interface^8,9^. Additional functions, for example, for chemical transformation or sensing applications^10,11^, can be added using core–shell particles or by decorating the microgels with inorganic nanoparticles^12,13^. Microgels are already used in several applications as viscosity modifiers and lubricants^14^, for CO_2_ capturing^15^ or 3D bioprinting^16^, as biocompatible additives and delivery vehicles^17^, sensors, or stimuli-responsive color-changing systems^18,19^.

Despite their popularity and widespread application, the internal structure of microgels is not well-understood. During the microgel synthesis, the degrees of freedom for the polymer strands are intentionally reduced by forming covalent cross-links between polymer chains. The chemical cross-linking itself is a nonequilibrium process, and it does not necessarily progress uniformly, which may lead to spatial fluctuations, gradients, and heterogeneities in the microgel particle architecture^20^. For a complete understanding of a microgel colloid as a material building block or a carrier, it is imperative to know the spatial distribution of polymer and cross-link densities on the nanoscale.

Initially, many studies assumed that microgels could be described simply as spherical, homogeneous gel particles, as discussed in ref. ^1^. Based on this assumption, the Flory–Rehner theory of polymer gels can be applied to make predictions about the elastic properties, and the swelling in a good solvent^21,22^. However, already more than 20 years ago, it was noticed that this scenario is highly unlikely, and that density gradients in the microgel conformation cannot be ignored^1,23^. Currently, the dominating picture is that standard microgel particles consist of a polymer gel core with a radially decaying cross-link density, and polymer dangling ends sticking out into the solvent. The dangling ends and the polymer strands in-between cross-links will thermally fluctuate, on nanometer-length scales, while the average spatial distribution of cross-links remains unchanged.

The inhomogeneous internal structure of microgels has profound consequences on the properties of microgel assemblies and their interactions. The cross-link density, and its spatial distribution, determines the elasticity and the swelling behavior, as well as the porosity of the gel network. The latter is vital for the uptake and release of small molecules^24^. The spatial distribution of cross-links and the properties of the interface control the onset of repulsive pairwise interactions between microgels, which is crucial for the jamming transition of densely packed microgels and also for their viscous losses^25^. In addition, the cross-links guarantee that microgels retain their structural integrity under shear, packing, and most other perturbations. The microscopic size and the hierarchical structure, from the atomic to mesoscopic-length scales, are at the core of microgel properties and their technological and fundamental relevance^26^. Progress toward a complete characterization of microgel colloids is, therefore, of great importance. In this perspective article, we will summarize the critical results obtained using conventional techniques, and discuss a set of innovative methods and emerging data.

## Microgel characterization with nanoscale resolution

**Fig. 2:**
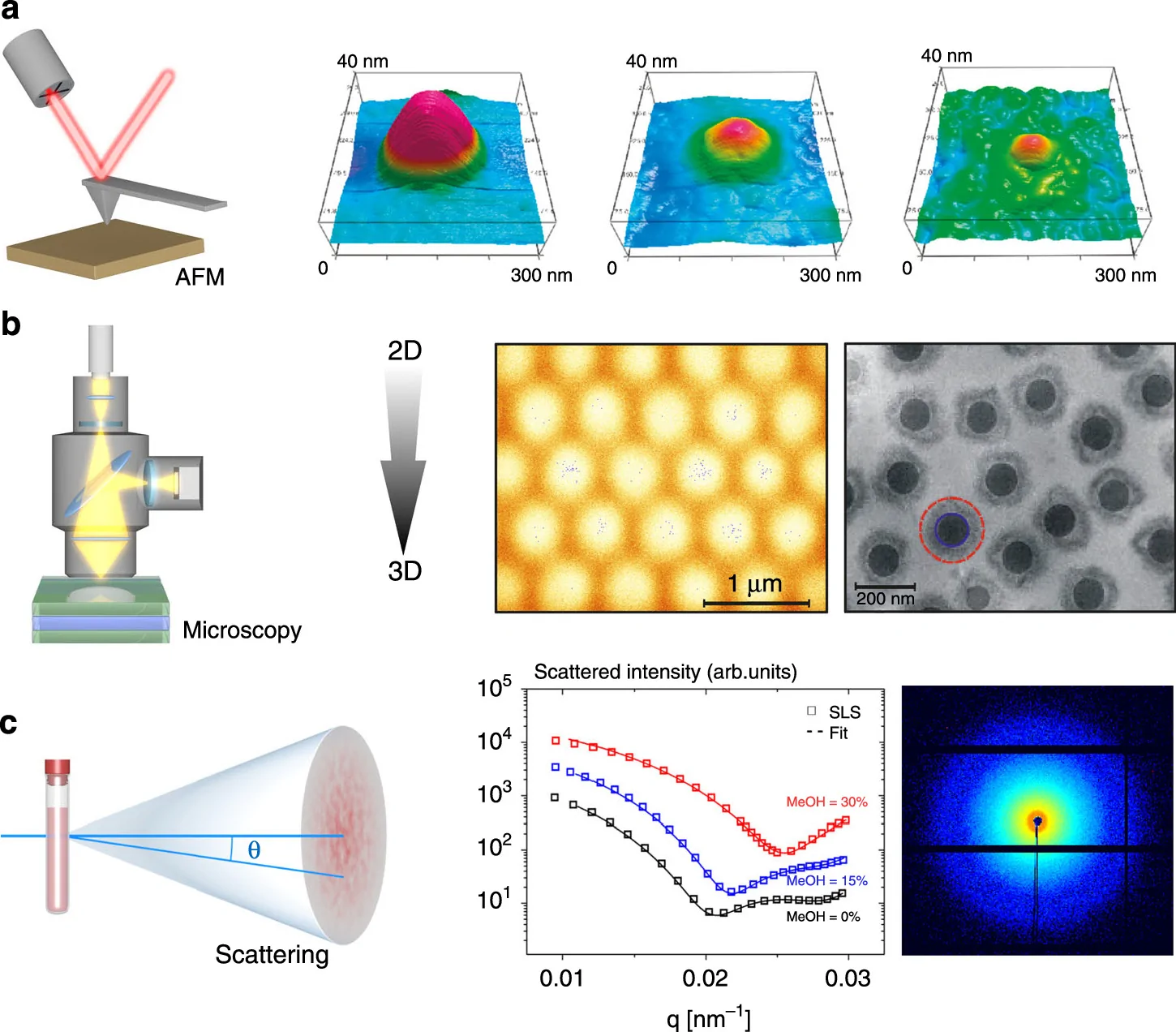
Conventional experimental techniques employed to characterize microgels and microgel assemblies. **a** Atomic force microscopy (AFM) is used to measure the shape and swelling of microgels adsorbed at a surface or interface^66,68,69^. Upon drying, the microgels flatten and acquire a lens-like shape. The AFM images on the right, adapted with permission from ref. ^67^ (Copyright American Chemical Society, 2010), show the erosion of a biodegradable microgel attached to a hydrophilic glass substrate after 0, 24,  and 434 h under ambient, dry conditions. **b** Microscopy: microgels have been studied using various microscopy techniques. Conventional light microscopy can probe microgels and their position, but not their morphology due to the lack of resolution. Using confocal laser-scanning microscopy (CLSM), the center position of microgels in dense assemblies can be tracked in situ in three dimensions. The bulk structure and phase transitions of microgel assemblies can be studied^26^. The first image shows an CLSM section of a fluorescein-dyed crystal assembled from 690-nm-diameter pNIPAM microgels, adapted with permission from ref. ^18^ (Copyright WILEY-VCH Verlag GmbH, D-69469 Weinheim, 2002). Cryogenic transmission electron microscopy (cryo-TEM) has the nanoscale-resolving power, but the sample preparation is difficult; rapid cooling may perturb the polymer conformation, and moreover the contrast is extremely low for the highly swollen networks. These difficulties have prevented widespread usage of cryo-TEM for the study of microgels. The second image shows a cryo-TEM micrograph of a 0.2 wt.% aqueous suspension of polystyrene core/pNIPAM shell particles, kept at room temperature, adapted with permission from ref. ^65^ (Copyright Springer Nature, 2008). The dashed circle indicates the hydrodynamic radius, as determined by dynamic light scattering (DLS). **c** Most of the experimental data on microgels stem from scattering, using light, neutrons, or X-rays. Scattering techniques provide information about the polymer density–density correlations in situ, and this information can be used to test models about the density profile, packing, interpenetration, and deswelling on a length scale from the nanometer to the micron range. The first image shows static light-scattering (SLS) curves obtained for submicron-sized  pNIPAM microgels suspended at low concentration in a water/methanol mixture for different methanol contents, reprinted from ref. ^58^ (Fig. 1a), with permission from Elsevier, Copyright (2016). The second image shows a small-angle X-ray scattering pattern obtained from a jammed suspension of hybrid microgels (pNIPAMs) with an embedded ellipsoidal hematite core, adapted from ref. ^127^ by permission of The Royal Society of Chemistry.

In the following sections, we will discuss the essential techniques that are applied to characterize microgels and concentrated suspensions of microgels, summarized in Fig. 2. We will emphasize new and emerging superresolution microscopy approaches that have already shed light on many critical open questions. We will highlight pathways and challenges toward a complete characterization of microgel colloids.

### Scattering techniques

When swollen, microgels are composed of a densely cross-linked core and a fuzzy corona. Already in 1994, Wu et al. postulated a core–shell-like structure based on the reaction kinetics^23^. It took until 2004 when Stieger et al. measured the particle form factor and derived a model for the radial polymer-density profile *ρ*(*r*)^27,28^. Neutrons, X-rays, or light can be used to measure the particle- form factor, depending on the size of the microgels, all suggesting qualitatively similar results for *ρ*(*r*)^29,30^. Dynamic light scattering (DLS) is routinely employed on dilute systems to derive the microgel hydrodynamic radius *R*_*H*_ from their random Brownian motion^29^. Small-angle neutron scattering has been used to assess the nanoscale correlation length *ξ* of the local polymer concentration fluctuations^22^. Scattering techniques can also be applied to hollow microgels as shown recently by Dubbert et al.^31^.

Scattering methods using X-ray, neutrons, and light do have the required nanoscale-resolving power and have thus been employed frequently^27,30,32,33^. However, they only provide information about radially averaged properties of an ensemble of particles, and therefore, information on a single-particle level is not accessible. Moreover, scattering methods work best if the constituents are composed of one material dispersed in a medium and are isotropic, i.e., they have a spherical shape, like a solid spherical particle or polymers in a good solvent, where only isotropic means can be measured. Scattering methods are less suited for anisotropic, deforming, densely packed, or interdigitated objects. In contrast to fluorescence microscopy, they lack specificity and perform poorly for hybrid, multicomponent particles, and microgels composed of chemically distinct entities. Nonetheless, refined scattering methods can overcome some of these shortcomings. Isotope labeling can be used to suppress structure factor contributions stemming from interparticle correlations, and reveal the single-particle-form factor, even at very high densities^28^. Using zero-average contrast (ZAC)–SANS, Mohanty et al. recently reported data on particle–particle interpenetration at elevated microgel densities^34^.

Two other methods that provide nanoscale, polymer-level, information on microgels, averaged over a large volume, are neutron spin–echo (NSE) and nuclear magnetic resonance (NMR). The methods can provide valuable information about the local dynamics and fluctuations that can then be linked to the heterogeneity of the gel network^35,36^.

### Light microscopy and nanoscopy

Compared with scattering techniques, optical microscopy offers essential advantages, such as access to single-particle information and chemical specificity using dye labels in fluorescent microscopy. Modern 3D-confocal laser-scanning microscopy (CLSM) became popular in the field of soft condensed matter in the 1990s^37^, and it has been applied to microgels soon after. In Fig. 2b, we show the results from a study by Debord et al. from the year 2002 on microgel photonic crystals^18^. The image reveals the strength and weaknesses of conventional optical imaging methods. It is easily possible to identify individual microgel particles with a diffraction-limited resolution of several hundreds of nanometers; however, all details on a particle or subparticle level are washed out. CLSM has been used repeatedly and successfully to study the center position of particles in dense assemblies^8,38,39^. Information on shorter length scales, until recently, could only be obtained from scattering techniques or electron microscopy, within the limitations of these methods.

The advent of super-resolution fluorescence microscopy (SRFM) techniques^40^ has opened up a whole new dimension for the characterization of soft polymeric materials^41,42^. Microgels and microgel assemblies have been one of the first systems that have benefited from these new developments. In contrast to solid particles, such as PMMA or polystyrene colloids^43^, for microgels, there has been an urgent need for improved resolution on sub-particle-length scales. Moreover, an active community has been working with dye-labeled microgels for two decades.

A broad range of far-field SRFM techniques has been developed in recent years. Some of these techniques, for example, linear structured illumination and 4Pi microscopy, do not attempt to beat the Abbe diffraction limit in microscopy and thus, by design, the resolution is limited. Still, these methods can deliver twofold improvements in lateral, and even more in axial, resolution, compared with optimized conventional microscopy using oil-immersion, high-numerical aperture objectives^44^. Another class of methods covers the so-called “true SRFM,” which can in fact overcome the diffraction limit. Examples include stimulated emission depletion (STED) microscopy^45^, photoactivated localization microscopy (PALM)^46^, stochastic optical reconstruction microscopy (STORM)^47^, and some more recent advanced implementations based on similar principles, such as (d)irect-STORM^48^, PAINT (point accumulation for imaging in nanoscale topography), and others^41,49^. Here, we will focus on these nanoscopy techniques^50,51^. All nanoscopy techniques employ a nonlinear optical principle on a fluorescence-labeled specimen, since a linear optical system cannot overcome the Abbe diffraction limit.

In STED, the fluorescent dyes are quenched using a so-called doughnut beam, and subsequently the excitation only remains in the center. The hole of the doughnut can in theory be made arbitrarily small, decreasing the optical resolution to the nanoscale. Already more than 10 years ago, Hell et al. reported an optical resolution of 5.8 nm (width of the point-spread function)^52^. More commonly, a resolution of 20–30 nm can be obtained, limited by the bleaching of the dye and its brightness. A disadvantage of conventional STED is that only the lateral resolution is improved, while the axial resolution along the *z*   direction remains diffraction-limited.

**Fig. 3:**
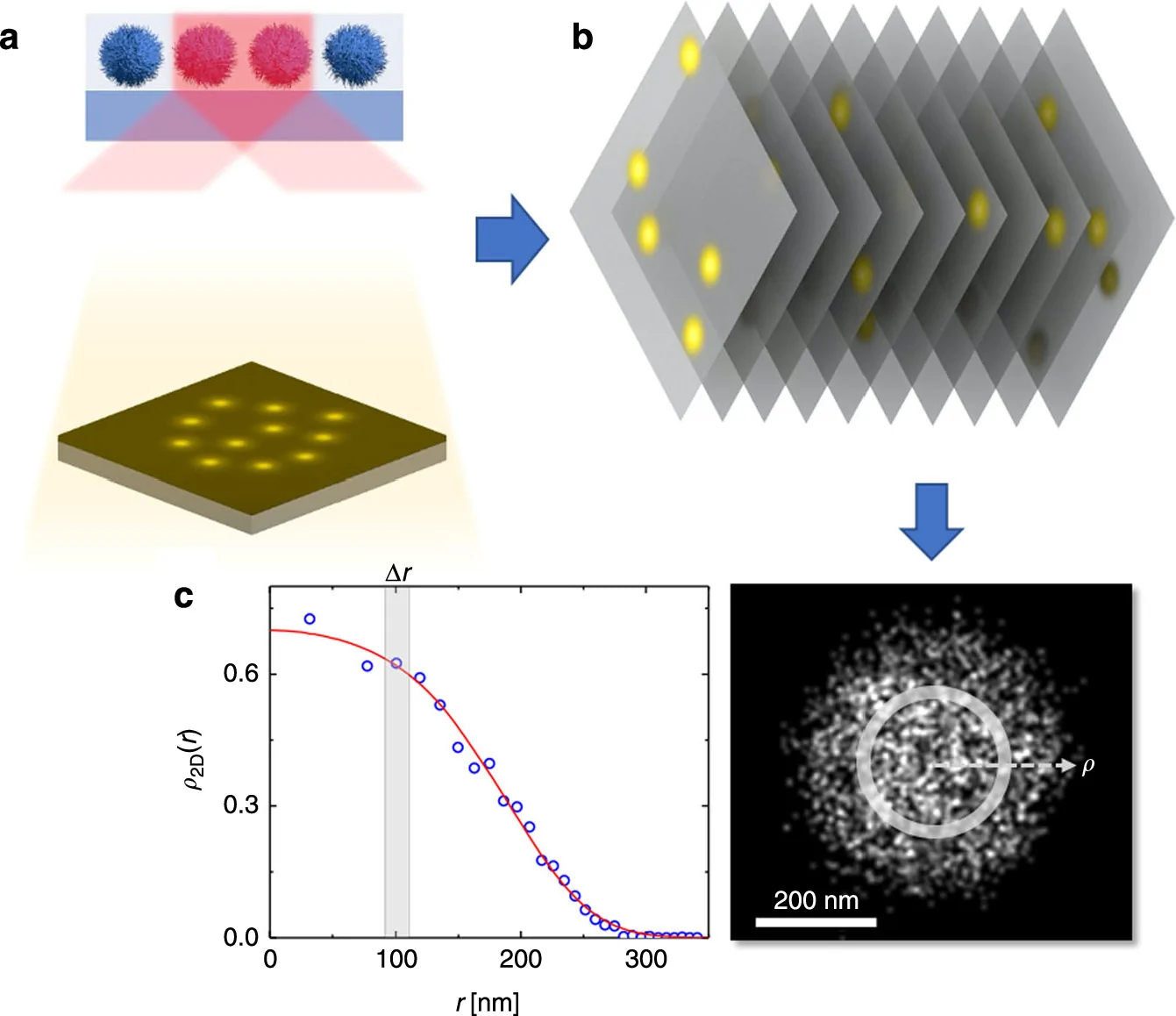
Superresolution fluorescent microscopy (SRFM). The most widely used method for soft-material imaging is single-molecule localization microscopy via direct STochastic Optical Reconstruction Microscopy (dSTORM)^48,57,58^. In dSTORM, a microgel can be labeled using conventional synthetic cyanine dyes such as Cy5 or dyes from the Alexa family. These dyes are then used as molecular photoswitches. **a** Highly inclined illumination of a microgel suspension with a laser beam (red shaded) to illuminate the dye-labeled particles within a few-μm depth, next to a glass coverslip, in blue. The inclined or evanescent field illumination reduces nonspecific fluorescence originating from the bulk of the sample. The illumination is provided by a powerful laser to achieve sparse fluorophore blinking, as shown in the image below. In contrast, another laser wavelength is used to tune the density of blinking molecules^58^. **b** For every dSTORM image, thousands of frames are acquired whereby each frame consists of thousands of fluorophore localizations, with typically thousands of photons detected per localization. The image acquisition takes up to several minutes, and therefore the microgels have to be immobilized on a glass substrate or trapped in a solid jammed or crystalline phase as in refs. ^25,110^. **c** Experimental result for a swollen microgel particle attached to a glass substrate and reconstructed by dSTORM in 2D with a 30-nm lateral resolution. From individual or ensemble-averaged images of this type, the density profile *ρ*_2*D*_(*r*) can be obtained by summing up the dye localizations in concentric rings around the center of mass. For spherical particles, *ρ*_2*D*_(*r*) can be converted into the three-dimensional (3D) density profile *ρ*(*r*) as explained in refs. ^58,86^. Alternatively, the $\rho (\overrightarrow{r})$ can be obtained directly in 3D as shown in Fig. 4. The dSTORM image and the single-microgel *ρ*_2*D*_(*r*) data have been adapted from ref. ^58^ (Fig. 2b; Supplementary Fig. S2), with permission from Elsevier, Copyright (2016). The solid red line shows the fit with Eq. (1) and *R*_*c*_ = 227 nm and *σ* = 40.5 nm.

PALM and STORM are single-molecule fluorescence techniques. At least initially, when both techniques were introduced, the fundamental difference between the two methods was that PALM uses photoswitchable fluorescent proteins (endogenously expressed), while STORM uses synthetic dye labeling^51^. For soft materials and polymers, only the latter is of concern, and in this paper, we will focus our attention on STORM. STED microscopy uses a deterministic method, meaning that an image is acquired in “one-scan,” while STORM and PALM are “stochastic” methods. For the last-mentioned, individual fluorophores are excited sequentially in time, which leads to dye “blinking”. Consequently, the single-molecule fluorescent signal is recorded without overlap of the diffraction-limited images (Fig. 3). From the accumulation of a larger number of positions of dye-labeled molecules, a superresolved image can be reconstructed, which does imply long acquisition times, on the order of tens of seconds or minutes, and thus in most cases, only static samples can be measured. It has been shown early on that three-dimensional information can be optically encoded, and optical imaging with a 20–30-nm lateral and 50– 60-nm axial resolution was reported on cellular structures more than 10 years ago^53^.

Another recent development is correlative microscopy, where two or more microscopy techniques are applied in tandem on the same sample to leverage the strengths of each method while offsetting their weaknesses^54^. 4Pi single-molecule switching nanoscopy (4PiSMSN)^55^ combines iPALM (interferometric PALM) and dual-objective 4Pi microscopy, and can achieve 10–20-nm isotropic resolution. Recently, Hell et al. combined the two SRFM principles of STED and single-molecule localization in a technique called MINFLUX, and they reported an ~1-nm precision^56^. MINFLUX is a relatively new technique that has not yet been applied widely to soft materials. Still, its advent is showcasing that an improved resolution down to the molecular scale is within reach. The combination of electron microscopy and SRFM is a powerful approach already used for imaging microgels decorated with inorganic nanoparticles, exploiting the differences in contrast in such hybrid systems^57^.

In biology, superresolution techniques have become very popular since the mid-2000s, and to this end, the Nobel Prize in Chemistry 2014 was awarded to Hell, Moerner, and Betzig. Nonetheless, it took several more years until superresolution techniques could get a strong foothold in soft materials science, and the application to microgels proved to be instrumental for this, as we will highlight in the next section^57,58^. A major advantage of STORM is the use of synthetic dyes that can be applied directly to polymeric materials or colloids, provided the dye labeling is done correctly, and the solvent conditions are suitable. Yet, the solvent puts some constraints on the material properties since the dyes require certain additives and an oxygen-scavenging buffer system^59^. Using solvent additives needed for best imaging results means that low ionic-strength conditions, e.g., for studies of ionic microgels^60^, cannot be easily accessed. We note in passing that some covalently bound switchable labels do neither require a particular buffer^61^ nor does the more recent PAINT approach (point accumulation for imaging in nanoscale topography)^41,49^. In practice, nanoscopy of individual solid particles has been performed early on as proof-of-principle for the method and to determine the optical resolution^45^, but the efforts usually ended there. Other soft-matter systems are either mobile, such as polymers or surfactants in solution, or difficult to dye-label appropriately, for example, polymer melts. Microgels offer an ideal platform in this respect since they are porous and spongy and can be dye-labeled throughout, while at the same time remain in contact with the solvent^40^.

SRFM can only provide accurate image information if the thermal density fluctuations are suppressed to length scales smaller than the desired resolution. This limit imposes sample-specific constraints, and for microgels, these are given by the correlation length *ξ* between cross-links in the range of several nanometers and the thickness of the fuzzy corona in the swollen state^22,32,62,63^. Nonetheless, even if the individual polymer molecule position cannot be measured, nanoscopy can still provide high-resolution images of the mean density in real space, similar to what scattering techniques of soft materials provide in reciprocal space. Moreover, the advantages of single-particle imaging and specific dye labeling can still be leveraged.

### Electron microscopy

Until recently, electron microscopy was virtually the only imaging method that could provide nanoscale resolution. To this end, conventional scanning electron microscopy is commonly applied to collapsed microgels in the vacuum^1^. Flattening of the soft microgels upon drying can lead to overestimates of the size. However, the shape change can also be exploited technologically since compressed microgels can be employed as microlenses^64^. Cryogenic transmission electron microscopy (cryo-TEM) is applied occasionally to image microgels in the hydrated and swollen state. Still, due to the low contrast, the image quality tends to be rather poor. Crassous, Ballauff, and coworkers have shown that the size of microgel-coated polystyrene particles can be extracted from cryo-TEM images^65^ (Fig. 2b). However, cryo-TEM has not yet revealed any quantitative information about the polymer density and its distribution inside the microgel.

### Atomic force microscopy

Atomic force microscopy (AFM) can probe the swelling and deswelling of microgels adsorbed to a surface^66–69^. However, the interaction of the AFM tip with the microgel and polymer indentation can complicate quantitative data analysis^66^. AFM does provide access to individual particle properties, and it can be applied to surface-adsorbed microgels, so it brings added value under these conditions^70^. It does, however, not provide direct, nanoscale-resolved images, and it certainly does not allow us to look inside the microgels or to study dense assemblies.

### Numerical modeling

A complete characterization of nanoscale objects is always a challenging task, and getting optimal results usually requires a combination of techniques. Numerical simulations also play an essential role since they can test specific effects explicitly, and can help to establish minimal models. Hard-sphere colloids, as well as different polymeric and soft-matter systems, have been modeled extensively and successfully^71^. Modeling and simulations of microgels, however, have lagged. Recently, first studies on mesoscale modeling of microgels have been reported^72,73^. The reasons for the limited results on microgel numerical modeling can be attributed to the complexity of the microgel network architecture, the still relatively poorly understood particle–particle interactions, as well as challenges in the understanding of the molecular origins of swelling and deswelling^74^. Despite these difficulties, significant efforts toward atomistic modeling are now underway. Pioneered by Zaccarelli and coworkers, atomistic models of the entire microgel were generated and successfully compared with experiments^75,76^ (Fig. 1b). While the models are atomistic, the force laws between the atoms still need to be adjusted ad hoc to reproduce experimental data. It remains an open question how predictive these models can be, and whether they can help to understand more complex architectures and dense assemblies.

## Characterization of microgels with superresolution microscopy

The properties of the polymer, the solvent, as well as the microgel architecture define how microgels act. On a more coarse-grained level, this can be translated into the elasticity of the individual microgels, microgel–microgel interaction potentials, as well the osmotic pressure balance between the microgel and the solvent phase. The latter depends on the solvent composition and ions released into the solvent by the microgels themselves. A thorough understanding of the individual microgel network characteristics, and in particular the polymer density $\rho (\overrightarrow{r})$ throughout the particle, will help us predict microgel behavior.

This perspective addresses in depth the properties of swollen microgels in solution. However, we will first briefly touch upon microgels at *T* > *T*_LCST_. Collapsed microgels are dense and homogeneous^30^; they behave very much like regular solid colloidal particles^77^. They feature, to a varying degree and depending on the synthesis parameters, charged groups at the surface, leading to double-layer repulsion and van der Waals (vdW) attraction, a situation that is often described by the DLVO interaction potential^78^. Depending on the degree of charging, the solvent ionic strength, and the vdW attractions, the collapsed microgel particles form a stable colloidal suspension; they flocculate or form a colloidal gel. In this paper, we will not address the aggregated particle assemblies and note that a rapid temperature quench of thermosensitive pNIPAM provides a way to prepare colloidal particle gels^79,80^. Temperature-induced aggregation of microgels is always reversible since in the swollen state, vdW attractions are weak.

### Microgel architecture and properties of isolated microgels

The vast majority of work is investigating microgels based on the monomer N-isopropylacrylamide (NIPAM) or its close derivatives^26^. The NIPAM molecule is widely used because the homopolymer displays a coil-to-globule transition at a lower critical solution temperature (LCST) *T*_LCST_ ≃ 32 °C, studied by Wu and Wang in 1998^81^. Free radical precipitation polymerization of the monomer is conducted in the presence of a cross-linker, typically N,N′ -methylenebisacrylamide (BIS), and initiated by potassium peroxodisulfate (KPS). Sodium dodecylsulfate (SDS) can be added as a stabilizer. During the synthesis, the cross-linker is consumed faster than the monomers, and therefore the core of the microgel particles is more densely cross-linked compared with the corona. Many variations of these synthesis protocols have been reported, with the aim, for example, to modify the response to stimuli, to create other architectures like multi-responsive-layer microgels^82^ or hollow microgels^31^. Microgels are usually dispersed in water and highly swollen under good solvent conditions at *T* < *T*_LCST_. The solvent quality can be changed such that the microgels deswell and eventually collapse, which is also known as the volume-phase transition (VPT). The latter can also be achieved by changing the pH^83^, solvent composition (adding, e.g., methanol)^58^, and by applying a high external osmotic pressure^84^.

Common neutral microgels feature a core–shell structure, and scattering data suggest a radial polymer-density profile *ρ*(*r*) of the type

1 $$\rho (r)=\frac{\rho (0)}{2}\ \,{\mathrm{erfc}}\,\left[\left(r-{R}_{c}\right)/\sqrt{2}\sigma \right],$$

with a core radius *R*_*c*_ and a shell thickness controlled by the “fuzziness” parameter *σ*^27,58^. It was shown that *R*_H_ ≈ *R*_*c*_ +  2*σ* provides a good estimate for the hydrodynamic radius^27,58^. More homogeneous microgels can be obtained by slowly adding the BIS cross-linker to the monomer solution, as described in ref. ^85^. Microgels synthesized, according to this recipe, have not yet been used widely. Bergman et al.^86^ speculated that homogeneous microgels might not be advantageous in practice, since the core–shell architecture is responsible for many of the unique and useful properties of microgels, for example, the soft onset of pairwise interactions. Here we will focus our attention on the standard type.

The characterization of microgels with scattering techniques is limited in two ways. First, the scattering of particles in solution records the Fourier transform of the pair-distance distribution function of the scattering density, which is a radially averaged signal in reciprocal space^87^. Possible particle anisotropies are very difficult to extract from a static scattering signal^29^. Second, scattering takes an ensemble average over a large number of particles. Polydispersity in terms of size, network morphology, but possibly also shape, is impossible to disentangle from scattering data. Therefore, attempts to obtain more detailed information about the microgel network morphology are futile.

**Fig. 4:**
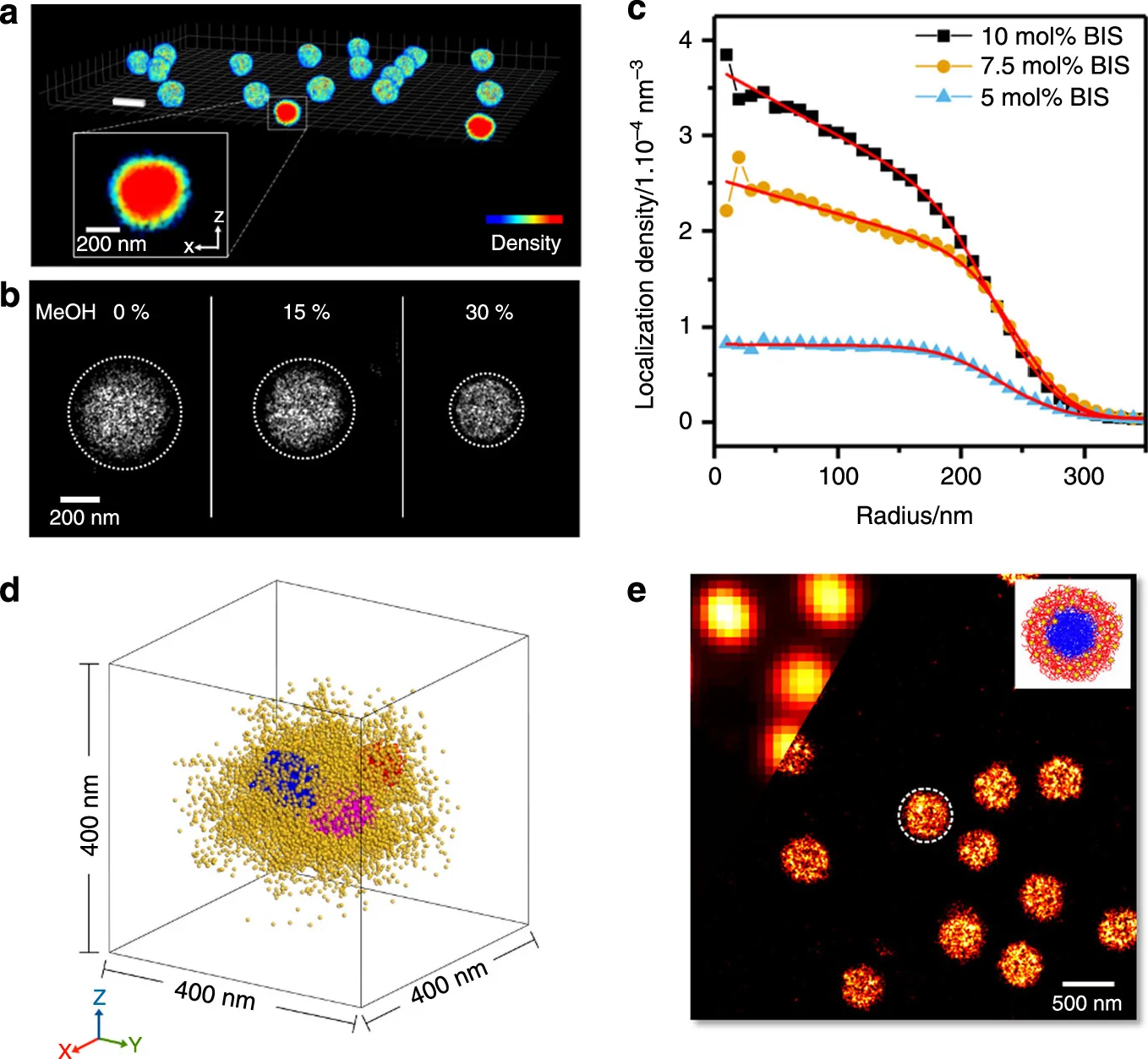
SRFM characterization of individual microgels. **a** 3D-dSTORM rendering of individual swollen neutral pNIPAM microgels (BIS 5%) in pure water, weakly adsorbed to a glass substrate (*T*  = 22 °C). The glass coverslips were treated with piranha solution (a 4:1 solution of sulfuric acid and hydrogen peroxide), which results in a highly hydrophilic surface (water contact angle 0° as reported in ref. ^128^). Inset: enlarged color-coded view of the internal density distribution. **b** Two-dimensional dSTORM of the same microgel particle and deswelling upon addition of methanol (MeOH). The dashed circles indicate the hydrodynamic diameter. Both panels adapted from ref. ^58^ (Fig. 2), with permission from Elsevier, Copyright (2016). **c** Density profile of dye localizations for similar neutral pNIPAM microgels suspended in pure water. The panel shows the results from a dSTORM study of microgels synthesized with different amounts of cross-linker (BIS = 5– 10%). For BIS concentrations above 5%, the data suggest a linearly increasing density toward the particle center. Reproduced from ref. ^86^ with permission from The Royal Society of Chemistry. **d** Single-molecule localization microscopy of near-spherical swollen microgel particles at 20 °C adsorbed to a polylysine-coated glass coverslip. The fluorescence localizations are determined for a small fraction of microgel cross-linker that has been dye-labeled. The (red, blue, and magenta)-color-labeled patches indicate clusters of increased cross-link density, while the localizations in the surrounding matrix are depicted in yellow, image reproduced with permission from ref. ^88^ (Copyright Royal Society of Chemistry, 2018). **e** dSTORM imaging of swollen pNIPAM-core–pNIPMAM-shell microgels in water deposited on a polylysine-coated glass coverslip. Inset: sketch of the particles. In the left upper corner, a part of the diffraction-limited image is shown. The dashed circle indicates the hydrodynamic diameter measured by DLS. The graphic was reproduced with permission from ref. ^57^ (Copyright American Chemical Society, 2016).

Testing the applicability of the fuzzy-sphere model, when more details of the network morphology can be resolved, has been the point of departure for the first STORM-superresolution study on microgels^58,86^. Conley et al. showed that pNIPAM microgels, attached to a hydrophilic substrate, possess a nearly ideal spherical shape (Fig. 4a), and that the core and shell polydispersity is highly correlated^58^. They reported density profiles of micron-sized, individual microgels synthesized with  ≃ 5% BIS, with a localization-density profile well described by the fuzzy-sphere model (Fig. 3c). More recently, Bergmann et al. showed that microgels obtained using higher cross-linker concentrations display unexpected deviations from the fuzzy-sphere model. They developed an interesting new method to resolve the network morphology. Utilizing the interaction of the freely diffusing fluorescent dye rhodamine 6G with the polymer network, they achieve indirect labeling, without altering the microgel chemical composition. Interestingly, they find that the polymer density in the core decays linearly before falling off exponentially in the corona (Fig. 4c). This additional weaker decay is only observed for BIS concentrations above 5%, and therefore had remained undetected in the first STORM study on microgels, see ref. ^58^.

Another recent SRFM study has focused on the properties of the microgel core. Karanastasis et al. report in situ 3D imaging, using 4PiSMSN, with a ~20-nm isotropic resolution^88^. This is likely the highest optical resolution reported for microgel optical imaging to date. They exploit the specificity of fluorescent labeling and directly tag part of the cross-linker with a dye. To this end, they obtain 3D maps of localizations of cross-link sites. They find that the cross-link density in the core is not homogeneous but rather clustered, as shown in Fig. 4d, with cluster sizes of tens of nanometers, embedded in a lower cross-link density matrix. Their findings agree with earlier proposals that the polymer cross-linking is structurally heterogeneous^35^. This direct observation of cross-link clustering inside the microgel core may help to explain the results by Bergmann et al., shown in Fig. 4c. The study also demonstrates the rapid progress with respect to resolution and specificity, in 3D real-space imaging of microgels with potential applications to many other soft-matter systems^42^.

A shortcoming of the study of Karanastasis et al. is the fact that the subset of dye-labeled cross-links, shown in Fig. 4d, is confined to a smaller volume than expected^88^. They find that Eq. (1) can describe the density distribution, but *R*_*c*_  + 2*σ* = 171 nm is much smaller than *R*_*H*_ = 330 nm^88^. The authors suggest that the dye-labeled cross-linker at 0.1% is consumed more rapidly compared with the BIS cross-linker at 2%. At least part of the difference in size could also be due to the lack of cross-linker in the corona, formed by the dangling polymer ends, and contributing to *R*_*H*_. In a parallel study, Siemes et al. also reported cross-linker density profiles using a novel diarylethene-based photoswitchable cross-linker with a highly fluorescent closed and a nonfluorescent open form. Interestingly, they report density profiles not in agreement with Eq. (1), a yet unexplained finding^61^. While the direct labeling of cross-linker molecules by these two groups is interesting and important, more work will be needed to unravel these contradicting results.

A better understanding of the network morphology and density distribution *ρ*(*r*) should, in principle, provide a basis to model the swelling behavior of microgels quantitatively. Despite the efforts made in the past^73,89^, this has not yet been achieved. More work is underway, using atomistic and mesoscale models to achieve this goal^72,75,76,90^. The challenge is to relate the measured density profile of the swollen microgel to the cross-linker gradient. Boon and Schurtenberger used a “nesting doll” model where each layer, with a specific cross-link density, is treated with the Flory–Rehner theory for swollen macrogels, and the energy functional of the entire microgel is minimized for a given cross-linker gradient^89^. However, for high cross-linker densities above 5% BIS, Bergmann et al. claim poor agreement between their experimental STORM data and this model. Recent experimental results by others, indicating cross-linker clusters inside the microgel core and heterogeneous domains within the network^61,88^, might provide clues for improvements of the nesting doll model. However, the treatment of the low-density brush-like outer corona might be challenging within the framework of the model^62,63,91^. The corona region can occupy up to 30–50% of the total volume of the swollen microgel^62,63,91^. Moreover, the outer shell dominates the onset of pairwise repulsive interactions when particles get into contact. The situation might be even direr for ionic microgels where Monte-Carlo simulations suggest that some of the dangling ends might stretch out to distances much larger than the core radius^92^. In a recent paper, Bergman et al. proposed that for ionic poly(NIPAM-co-acrylic acid) microgels, the corona and cross-linked core swelling can be considered as two separate entities^93^. Currently only DLS is able to reliably quantify the corona swelling, by measuring the hydrodynamic radius *R*_*H*_. Due to the extremely low density of the corona, both scattering and microscopy techniques have been unable to provide quantitative data. Selective dye labeling and improved contrast using better, brighter, and more stable dye markers hold promise to get a better grip on corona properties in future SRFM studies^94^.

The field of nanoscopy of microgels is still relatively new, and many microgel systems and questions around microgel properties have not yet been addressed. For example, it would be fascinating to image ionic microgels^39,95^, also under different solvent conditions and ionic strengths. Equally, ultralow cross-linked^96^ and very small microgels^97^ would benefit from a better characterization of their morphology. We expect that such studies will be conducted within the next few years, providing more high-quality data as an input for theoretical models and simulations.

### Functional microgels and microgel–nanoparticle hybrids

Most of the recent advances in nanoscale characterization have been targeted toward simple, radially isotropic microgels. Here, we would like to point toward not only experimentally more challenging but also highly exciting applications of nanoscale imaging, such as functional microgels, for example, microgels with certain comonomers, polymer–nanoparticle hybrids, Janus particles, or microgel-based colloidal molecules^98–101^. A comprehensive discussion of functional microgels would be beyond the scope of this paper and can be found elsewhere^12^. However, the power of SRFM when applied to such functional systems has already been demonstrated by Gelissen et al. in one of the first STORM studies on microgels^57^. They successfully combined SRFM and electron microscopy (TEM) to unravel structural details of microgel core–shell particles (Fig. 4e), and microgels decorated with gold nanoparticles. Experiments of this type set the stage for the future development toward new levels of resolution and specificity, through multicolor dye labeling, based on SRFM^54^.

### Adsorbed microgels on functionalized surfaces

**Fig. 5:**
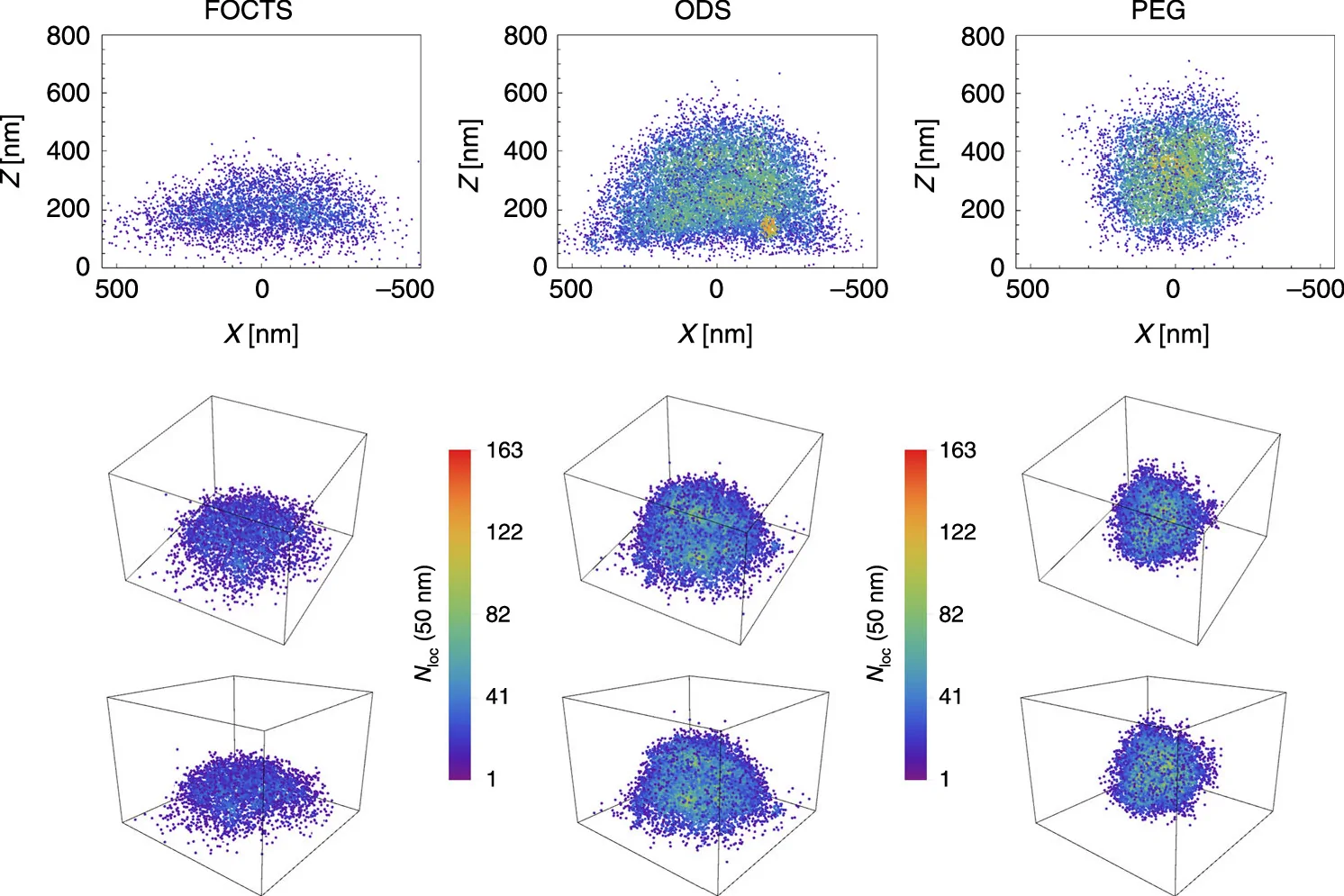
Adsorbed pNIPAM microgels on surface-functionalized glass imaged with three-dimensional dSTORM^103^. The upper graphs show a vertical section (*y* plane) of the localization-density distribution. The lower graphs show three-dimensional renderings at two different viewing angles. The functionalization with hydrophobic trichloro-(1H, 1H, 2H, 2H-perfluorooctyl)silane (FOCTS), or n-octadecyltrimethoxysilane (ODS), results in a flattened lens-like shape, while on surfaces functionalized with hydrophylic O-(2-aminoethy)polyethylene glycol (PEG), the microgel particles retain their spherical shape in agreement with the data reported previously in ref. ^58^ and shown in Figs. 3 and 4. The figure was reproduced with permission from ref. ^103^ (Copyright American Chemical Society, 2019).

Microgels are frequently studied at interfaces or deposited on a glass surface for analysis, both in the wet and the dry state. In the first SRFM study of microgels^58^, as well as subsequent work^57,86,88^, the microgels were attached to a glass substrate and immobilized. Based on 3D-dSTORM (Fig. 4a), Conley et al. reported microgels of spherical shape when attached to a hydrophilic glass substrate. The 3D images were, however, not analyzed quantitatively, and the calibration of the *z* axis in 3D-dSTORM is delicate^102^. The possibility of calibration errors thus leaves some ambiguity about the microgel sphericity in this study. This question was recently addressed by Alvarez et al., also using 3D-dSTORM. They studied the deformation and spreading of microgels on functionalized surfaces in an aqueous environment^103^. As shown in Fig. 5, they found that on hydrophobic surfaces, microgels spread and acquire a pancake-like shape, while on hydrophilic surfaces, microgels retain their spherical shape. The systematic data by Alvarez et al. thus confirm the original results of Conley et al., but they also show that one has to be careful with such studies.

### Effective pairwise interactions between microgels

The local particle–particle interaction potential and the spatial distribution of microgel particles determine the properties of microgel-based materials at *T* < *T*_LCST_. For neutral particles, the compressibility of the microgel network and corona defines the pairwise particle interactions^25,91^. The contributions due to van der Waals attraction forces are usually weak  ≪*k*_*B*_*T* due to the low polymer density in the swollen state. For ionic microgels, additional contributions due to the overlap of the ionic clouds come into play^78^. For simplicity, we restrict our discussion to neutral microgels where possible contributions of the DLVO type, both attractive and repulsive, are small or negligible. Therefore, we only need to consider repulsive forces that arise from elastic deformations of the microgel. The most common way to address this problem is to model repulsive interactions with a Hertz contact potential. This approach is equivalent to treating the soft microgel particles as homogeneous elastic spheres^104^. The model can be generalized to a multi-Hertzian potential, i.e., a multilayer structure of coupled Hertzian potentials, to take account for the fuzzy polymer-density profile^91^. Other models assume a homogeneous soft core covered by a polymer brush formed by dangling ends^62,63^. Such approaches can fit the experimental data well. They do, however, require several adjustable parameters that have not yet been linked quantitatively to the fundamental properties of the polymer network and its morphology. Based on the new and emerging knowledge from SRFM, we believe that it should soon be possible to improve this situation and attain a better quantitative understanding of microgel–microgel pairwise interactions. Efforts are also underway in our laboratory, for example, to directly measure pair-interaction potentials using optical tweezers, inspired by recent measurements on colloidal star polymers^105^.

### Structure and morphology of densely packed microgel-based materials

The colloidal phase behavior of microgels, and charged and neutral soft spheres in general, has been the subject of many studies^95,106–108^. For a sufficiently monodisperse pNIPAM microgel suspension that is slowly cooled below *T*_LCST_, we expect crystallization at an effective volume fraction *ζ*  ≳ 0.5. For a polydispersity greater than *δR*_*H*_/*R*_*H*_ ~ 0.1 or for a rapid quench, crystallization is suppressed or slowed down, leading to an amorphous solid phase^108^. In an experiment, it is not always obvious which phase has been obtained. Typically, for smaller microgels, the crystal phase shows iridescence^109^, whereas, for larger, micron-sized, microgels, small-angle light scattering (SALS) can detect the presence or absence of sharp Bragg peaks^110^. In both cases, the particles eventually touch and, upon reaching higher values of *ζ* > 0.7, have to adapt their morphology. A number of things can happen. Microgels can deform like elastic spheres^2,8,106^, they can interpenetrate, or they can shrink^28,111^. Usually, all three processes may proceed at the same time, such that the total free energy reaches a local or global minimum.

**Fig. 6:**
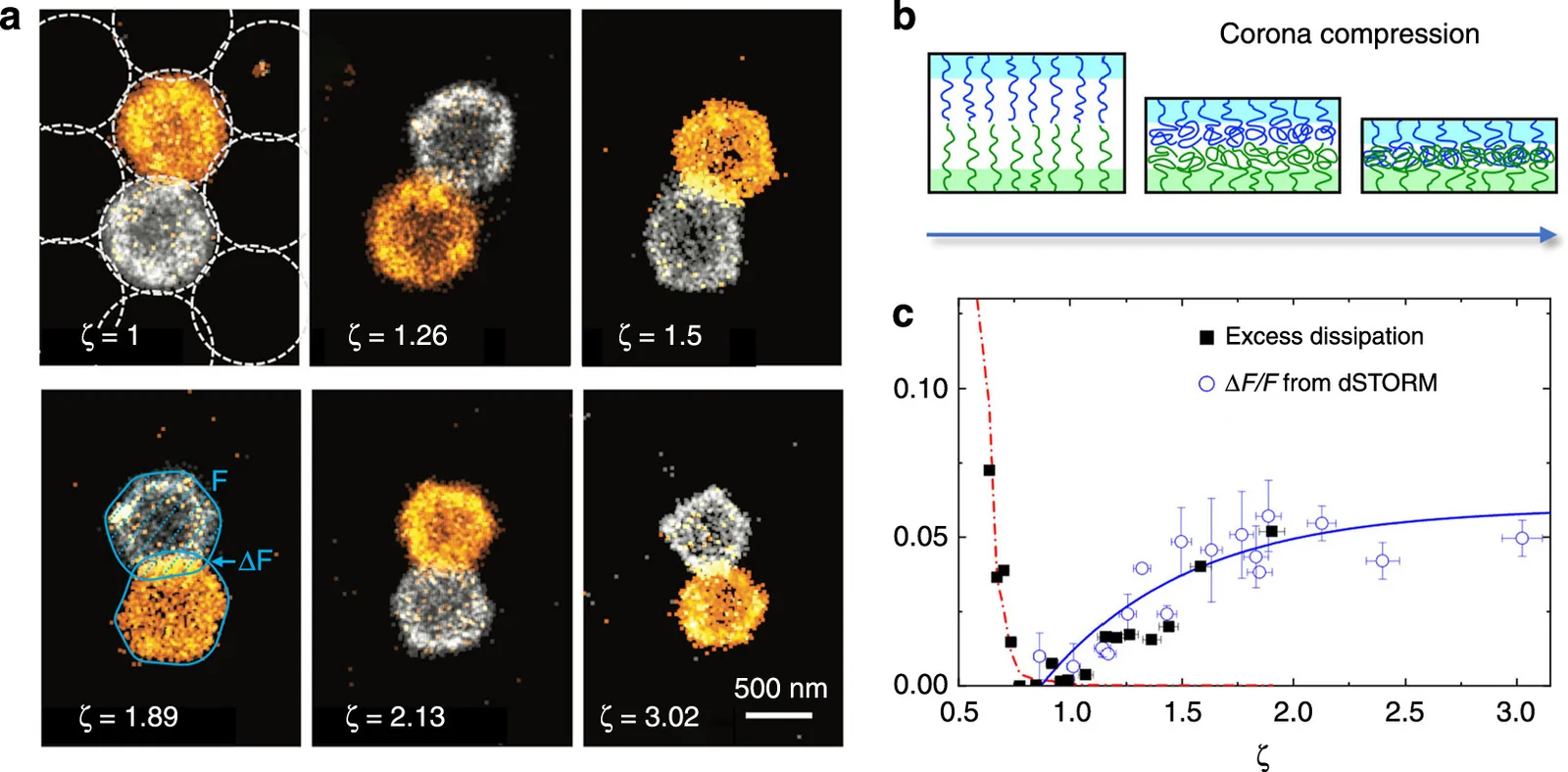
Densely packed suspensions of neutral microgels: deformation, interpenetration, and compression. **a** Two-color 2D dSTORM of dye-labeled microgels embedded in a matrix of unlabeled microgel particles, indicated by dashed circles in the left upper panel. *ζ* is the effective volume fraction. SRFM imaging of specifically labeled microgel pairs reveals partial interpenetration, faceting, and finally isotropic compression, leading to a decreasing global size. Contour lines are shown for *ζ* = 1.89 to illustrate the overlap area Δ*F* relative to the total area *F* of a particle for the image section shown. Image adapted from ref. ^110^. **b** Interpenetration mechanism at the particle–particle interface suggested in ref. ^25^ based on the dSTORM data. **c** Evidence that the dissipative behavior, expressed by the excess dissipation $\tan {\delta }_{\mathrm{exc}}={G}^{\prime\prime}/{G}^{\prime}-{\left({G}^{\prime\prime} /{G}^{\prime}\right)}_{{\mathrm{min}}}$ at *ω* ≃ 1 rad/s, scales with the overlap area 0.4 × Δ*F*/*F*. The solid blue line is a guide to the eye, and the red dash-dotted line denotes $1/{G}^{\prime}$. These results suggest that information from nanoscale SRFM imaging can be directly used to model viscoelastic properties. Panels (**b**) and (**c**) were adapted from ref. ^25^.

Recent SRFM experiments have been instrumental in identifying the various responses of microgels upon packing^110^. These experiments also highlighted the enormous potential of SRFM techniques applied to microgels and soft matter in general since they illustrated how qualitatively new information about bulk polymeric systems can be obtained. In the study reported in ref. ^110^, submicron-sized, highly swollen pNIPAM microgels were synthesized with  ≈5% BIS cross-linker. Dense suspensions were prepared above *T*_LCST_ and then quenched to 22 °C, well below the LCST. Effective volume fractions from *ζ* = 0.7 to *ζ* ≃ 3 were investigated. We may note in passing that overpacking (*ζ*  > 1) cannot be reached using incompressible soft colloids, for example oil droplets in a dense emulsion^112^. The dSTORM experiments revealed that the packing progresses in different stages. In Fig. 6a, we reproduce typical two-color dSTORM images for several packing fractions. Initially, the fuzzy corona is compressed onto the core, resulting in rather homogeneous soft spheres at *ζ* = 1. Next, the particles deform, facet, and weakly interpenetrate, thereby reducing spatial density fluctuations. Eventually, no further interpenetration takes place, prevented by the network topology. Once this stage is reached, the spatial polymer distribution is nearly homogeneous on length scales larger than the network correlation length *ξ*, as confirmed by the vanishingly low light-scattering contrast^110^, and the microgels shrink when increasing the concentration further.

The neutral microgels studied in refs. ^25,110^ are of the most common pNIPAM based type, with a radius of *R*_*H*_ ≃ 500 nm and  ≃5% BIS, and therefore the results are representative for many microgel studies carried out in the past. However, a plethora of other types of microgels have been discussed as well, and the characteristics can vary substantially. Differences can arise due to different polymer chemistry, size, cross-link density, and charges. We expect SRFM to play an essential role in disentangling the packing behavior of dense microgel suspensions for a number of microgel types and under different conditions. Potentially, SRFM can also be applied under out-of-equilibrium conditions, such as macroscopic shear deformation. Still, we need to keep in mind that it takes minutes^25^, rather than seconds, to record a single dSTORM image. Recently, progress in localization microscopy has been reported pointing toward second-scale image acquisition in biological applications, but the performance of these approaches has not yet been tested on soft materials^113^.

A fascinating open question, concerning dense microgel suspensions, is the spontaneous deswelling by counterion clouds^114^. Earlier work by Fernandez-Nieves et al. showed that microgels deswell when subject to a high external osmotic pressure, by adding dextran to the solution^84^. Scotti et al. suggested that the counterions released by the microgels themselves could lead to a reduction in particle size with increasing microgel concentration. From this, they conclude that in bimodal or polydisperse mixtures, larger particles selectively shrink. As a consequence, uniform shrinkage may suppress or delay the liquid-to-solid transition and facilitate crystallization^115^. If and under which conditions such deswelling may occur is not entirely clear and has been debated^25,92,116^. SRFM and scattering techniques will play a pivotal role in settling this question.

### Rheology of microgel-based materials

Microgels possess remarkable properties, such as a very low mass density, porosity, softness, and responsiveness to external stimuli. The nano- and microscale properties of microgels are widely exploited in bulk formulations where microgels act as viscosity modifiers and lubricants^2,14^. Microgels have also become popular model systems in experimental studies of soft and adaptive colloids in highly concentrated and jammed suspensions^3,106,117–121^.

The rheology of microgels is especially interesting because it is susceptible to the particle morphology, swelling, and particle–particle interactions. Mattsson et al. reported that some microgel systems can be overpacked up to ninefold (*ζ* ≈ 9), and exhibit viscous flow properties over the entire range^122^, while other systems, like the one discussed in Fig. 6, jam at *ζ* ≈ 0.6 like elastic spheres and form fairly strong solids for *ζ* > 1^25,62,109,110^ with elastic moduli exceeding 10^3^ Pa. Clearly, the microscopic properties of the microgels must be responsible for this fundamentally different macroscopic behavior. Ikeda et al. discussed the interplay between the entropically driven glass transition and the buildup of deformation energy due to contact formation^108^. Quite generally, the size, the softness, and the architecture of microgels play an important role^97^. However, in many previous studies, the microgels under investigation have not been characterized in much detail. Modern SRFM techniques provide an opening here to make progress toward a complete characterization of individual microgels, dense microgel-based systems, and consequently reach a better understanding of their rheological properties. In the following section, we present an example of such an attempt to use the microscopic knowledge obtained from SRFM for the interpretation of rheological data.

Oscillatory shear experiments on neutral, submicron-sized pNIPAM microgels, some dating back to the 1990s^109^, have reported a sharp onset of the low-frequency (*ω* ~ 1 rad/s) elastic modulus ${G}^{\prime}$ at random close packing or “jamming,” followed by a more gradual linear increase for *ζ* > 1^25,107^. Some experiments suggest a critical behavior at *ζ*_c_ ~ *ζ*_J_ ~ 0.6, characteristic of a jamming transition^62,104,107,109^. To account for the increase in the elastic modulus ${G}^{\prime}\left(\zeta \right)$, different elaborate models have been proposed^62,63,91,104,107–108^. A quantitative assessment of the microscopic packing behavior, a key ingredient for all modeling attempts, has been missing however. In particular, it was unknown to which extent microgel particles facet or interpenetrate, and if and when they may spontaneously shrink. We recently established such a relationship between the nanoscale structure and the rheological properties, using two-color dSTORM microscopy^25^. The packing evolution, shown in Fig. 6a, suggests that for *ζ* < 1, pairwise interactions are dominated by the compression of the microgel corona. For *ζ* > 1, we observe the faceting and subtle interpenetration of fairly homogeneous soft spheres followed by the shrinkage or isotropic compression of the individual microgels. In tandem, for *ζ* > 1, the low-frequency elastic shear modulus ${G}^{\prime}$ increases linearly with *ζ*. From this, we conclude that for *ζ* > 1, the elastic modulus is set by the jamming and compression of homogeneous microgel spheres. At lower concentrations, the corona compression dominates that of the pairwise interactions between microgels, and therefore ${G}^{\prime}(\zeta )$ rises much more rapidly once particles are touching, for *ζ* ≳ 0.6. Similar results for ${G}^{\prime}(\zeta )$ have been reported recently by Pellet and Cloitre^107^. We also looked at losses under oscillatory shear. For weak corona compression *ζ* < 1, the loss modulus *G*^*″*^(*ω*) matches the trend expected for other soft spheres with lubrified interfaces, such as emulsion droplets. At higher densities however, the contribution of viscous losses increases substantially. The latter can be quantified by the ratio of the loss modulus and the storage modulus at low frequencies, $\tan \delta ={G}^{\prime\prime}/{G}^{\prime}$. Interestingly, we find that the degree of overlap between adjacent microgels measured by dSTORM, Δ*F*/*F*, is directly proportional to the excess dissipation, as shown in Fig. 6c.

## Conclusions and outlook

There have been tremendous advances toward a complete characterization of microgel colloids over the last few years. The emerging superresolution microscopy techniques have primarily driven this progress since 2016. While the first experiment focused on validating the method, more recently, we have seen many fascinating examples of how SRFM microscopy can improve our understanding of microgels and microgel-based materials. More studies of this kind will follow, addressing different types of microgels and microgel-based materials, looking at encapsulation and release of pharmacological substances, flavor, or fragrances^24,123^.

At the same time, it remains essential to question each time the accuracy and meaningfulness of fluorescence microscopy data, notably when pushing the resolution limit toward the 10-nm scale. Recent work suggested cross-link clustering in the microgel core and deviations from the fuzzy- sphere model, as discussed in Fig. 4. Likely, these results point toward interesting and emerging new phenomena that will help us to better understand the structure, morphology, and swelling of microgels. Nevertheless, it will be important to corroborate the results by reproducing them in other labs, ideally with a different dye or labeling chemistry. Moreover, it will also be important to carefully consider the influence of polydispersity, and the way the data are analyzed in SRFM. Often, image sections are recorded, which are projections from an image slice onto a 2D plane, from which an ensemble average is calculated, and sometimes these results are transformed back to 3D^58,86^. In addition, the finite optical resolution and possible imaging artifacts have to be considered. Until now, most SRFM images have been recorded for adsorbed microgels or microgels located within a few-particle diameters next to a glass surface. As recently shown and discussed in Fig. 5, the presence of an interface can strongly influence the microgel shape, and thus, care has to be taken when interpreting such data.

In parallel to the rapid advances in microscopy, there has also been substantial progress with other characterization techniques. Modern scattering techniques can reveal important information, and can be applied to large bulk systems. Due to the possibility to perform contrast variation, neutron scattering is very powerful^34^. Many other techniques remain very useful, and there is certainly a tremendous potential for correlative approaches. The combination of SRFM, electron microscopy, scattering, and other methods seems to be ideal for addressing many of the open questions about microgels.

In this paper, we have focused our attention almost exclusively on the structural aspects of microgels. Most of the recent progress in superresolution microscopy has been made in this arena. While some SRFM techniques may also allow dynamic studies, there has been little research activity to date. Localization methods such as STORM are very slow, and it will be challenging to reach sub-second time resolution without compromising on image quality and resolution. Other SRFM techniques, such as STED, are faster, but to date, have not been applied to microgels. Time-resolved scattering techniques^124^, inelastic neutron scattering^125^ and neutron spin echo (NSE)^126^, diffusing wave spectroscopy^62^, oscillatory shear^25,107^, NMR^35^, dynamic AFM^70^, and other conventional techniques, however, can provide a plethora of information about the motion and internal relaxation of microgels, from the nanoscopic to the mesoscopic scales. In the future, we expect more studies that will combine modern high-resolution structural characterization methods with advanced dynamical measurements.
